# A new genus and species of pinworm (Nematoda, Oxyuridae) from the gray mouse opossum, *Tlacuatzin canescens*

**DOI:** 10.1051/parasite/2019054

**Published:** 2019-08-21

**Authors:** F. Agustín Jiménez, Juan Manuel Caspeta-Mandujano, Sergio Albino-Miranda

**Affiliations:** 1 Department of Zoology, Southern Illinois University Carbondale IL 62901-6501 USA; 2 Laboratorio de Parasitología de Animales Silvestres, Facultad de Ciencias Biológicas, Universidad Autónoma del Estado de Morelos Av. Universidad No. 1001, Col. Chamilpa CP 62210 Cuernavaca Morelos México; 3 Red de Biología y Conservación de Vertebrados, Instituto de Ecología A. C. Carretera Antigua a Coatepec 351, El Haya, Xalapa 91070 Veracruz México

**Keywords:** *Tlacuatzoxyuris simpsoni*, *Tlacuatzin canescens*, Great American Biotic Interchange, Endemism, Oxyuridae, Scientific collections

## Abstract

*Tlacuatzoxyuris simpsoni* n. gen. n. sp. is described from the cecum of the gray opossum, *Tlacuatzin canescens*, a species endemic to the deciduous dry forest of Mexico. The digestive tracts of four specimens were examined for parasites; three of these were archived in the American Museum of Natural History and one was a live capture. Relative to the other four monotypic genera of pinworms known to infect opossums, the new genus is diagnosed on the basis of a round cephalic plate with a semicircular stoma surrounded by a rim. In addition, males feature a prominent cephalic vesicle not fully developed in females, accounting for sexual dimorphism. The new species includes small worms that feature a conspicuous, not reticulated cephalic vesicle and semicircular stoma and lateral alae with two crests. In addition, the postcloacal cuticle of males features a small area with ornamentation between cloaca and submedial papillae. Finally, both spicule and gubernaculum are relatively short. Although the eggs of *Tlacuatzoxyuris* n. gen. are unknown, the conspicuous differences in traits used in the diagnosis of genera prompted us to propose a new genus for the new species. This is the first species of Oxyuridae reported in mouse opossums outside South America, and the fifth species of the family occurring in didelphimorph marsupials. This is an example of the usefulness of documenting the diversity of parasites associated with this unique clade of mammals through the examination of preserved tissues.

## Abstract (fr)

*Tlacuatzoxyuris simpsoni* n. gen. n. sp. est décrit à partir du caecum de l’opossum-souris gris *Tlacuatzin canescens*, une espèce endémique de la forêt sèche à feuilles caduques du Mexique. Les appareils digestifs de quatre spécimens ont été examinés pour rechercher des parasites ; trois d’entre eux étaient archivés au Musée Américain d’Histoire Naturelle et un provenait de capture. Par rapport aux quatre autres genres monotypiques d’oxyures connus pour infecter les opossums, le nouveau genre est diagnostiqué sur la base d’une plaque céphalique ronde avec une stomie semi-circulaire entourée d’un rebord. De plus, les mâles présentent une vésicule céphalique importante non développée chez les femelles, ce qui explique le dimorphisme sexuel. La nouvelle espèce comprend des vers petits, présentant une vésicule céphalique bien visible, non réticulée, une stomie semi-circulaire et des ailes latérales avec deux crêtes. En outre, la cuticule postcloacale des mâles présente une petite zone ornementée entre le cloaque et les papilles sous-médiales. Enfin, le spicule et le gubernaculum sont relativement courts. Bien que les œufs de *Tlacuatzoxyuris* n. gen. soient inconnus, les différences marquées dans les traits utilisés dans le diagnostic des genres nous ont incités à proposer un nouveau genre pour la nouvelle espèce. Il s’agit de la première espèce d’Oxyuridae signalée chez les opossums-souris en dehors de l’Amérique du Sud et de la cinquième espèce de la famille chez des marsupiaux didelphimorphes. Ceci est un exemple de l’utilité de documenter la diversité des parasites associés à ce clade unique de mammifères grâce à l’examen de tissus préservés.

## Introduction

Marsupials are a typical component of the Neotropics [28, 30, 31]. In the Americas, this group of mammals includes three families in three orders of which Didelphidae (Didelphimorphia: Didelphidae) contains roughly 110 species of small to medium-sized marsupials [11, 23, 27, 32]. These diversified into arboreal, scansorial, terrestrial, and semiaquatic habits [4]. The continuous investigation of the basic biology, distribution and diversity of these marsupials has allowed researchers to document the isolation of some populations, to establish the geographic and taxonomic limits for several genera, and to generate evidence to formally describe new didelphimorph taxa [3, 14, 16, 29].

Perhaps because of their ubiquitous nature and high abundance, most helminthological records are centered in four species of medium-sized opossums [1, 19, 25], yet very few helminth taxa had been described from small sized marsupials of arboreal habitats [2, 5, 7, 9, 13, 17, 18, 20, 22, 24]. Among those, four monotypic genera of nematodes infecting arboreal and terrestrial mouse opossums have been described in South America. These include *Neohilgertia venusti* Navone, Suriano & Pujol, 1990, from *Thylamys venustus*; *Didelphoxyuris thylamisis* Gardner & Hugot, 1995 from *Thylamys elegans* and *Thylamys venustus*; *Monodelphoxyuris dollmeiri* Guerrero & Hugot, 2003 from *Monodelphis emiliae*, and *Gracilioxyuris agilisis* Feijó, Torres, Maldonado & Lanfredi, 2008 from *Gracilinanus agilis* and *Marmosa paraguayana* [8, 12, 15, 21]. Most of these species are characterized by their relatively short tail and by the presence of numerous mamelons on the ventral side. The criteria to differentiate each of these monotypic genera include the configuration of the stoma or buccal opening, presence of a cephalic vesicle, as well as the ornamentation of the egg shell. The stoma in the four known species is an important element in the diagnosis of these taxa; it can be surrounded by three “lips”, which cover 3–4 pharyngeal teeth. The cephalic vesicle is either absent, or thick, with conspicuous internal vesicles in the females of *D. thylamisis*. Finally, the eggs have been characterized as either operculated or non-operculated, and the eggs of *G. agilisis* feature three longitudinal ridges which are used in the diagnosis of the genus.

Herein, we describe a new species of pinworm found in the large intestine of the gray opossum, *Tlacuatzin canescens* (Allen, 1893) in the dry forest of southern Mexico. Since it shows clear differences in the configuration of the cephalic vesicle and the stoma with the other four monotypic genera, we propose a new genus to include it. This is the fifth oxyurid species identified in didelphid marsupials and the first one occurring north of the basin of the Amazon river. The finding of this nematode in a marsupial north of the Panama isthmus has implications relative to the dispersal of fauna of South American origin into the North American tectonic plate, which is part of the reciprocal biotic exchange among the two landmasses of the New World known as the Great American Biotic Interchange.

## Materials and methods

A single gray mouse opossum was hand trapped in the locality of Cruz Pintada: Morelos: Mexico (18°27′58″N, 99°2′16″W, 1016 m) on 10 June 2010. The individual was humanely euthanized and examined for parasites 12 h after trapping, following methods approved by the Institutional Animal Care and Use protocol 09-026 (Assurance Number A-3078-01). Specimens resulting from this examination were fixed in 4% buffered formaldehyde and subsequently transferred to 70% ethanol for storage. In addition, the digestive tracts of three gray opossums and three Mexican mouse opossums (*Marmosa mexicana* Merriam, 1897) collected in the isthmus of Tehuantepec were requested from the American Museum of Natural History (AMNH) (New York, NY). These tissues were rinsed and cut open, all removed metazoans were washed and saved in 70% ethanol. Collection numbers and locations for these specimens are detailed in Table 1. All 15 pinworms found were cleared in temporary mounts of lactophenol or glycerine. All measurements are given in micrometers and detailed in Table 2; for each character, the range is given first, followed by mean and coefficient of variation [26]. After clearing, two specimens, a male and a female, were washed and then dehydrated progressively in a graded ethanol series, dried to a non-liquid state by critical point drying using CO_2_, attached to an SEM stub, and sputter coated with gold palladium. Specimens imaged with SEM were exposed to a beam of 2–10 kV on a FEI Quanta 450 Field Emission Scanning Electron Microscope (FEI, Hillsboro, Oregon). Specimens of *D. thylamisis* Gardner & Hugot, 1995; *Monodelphoxyuris dollmeiri* Hugot & Guerrero, 2003, and *Neohilgertia venusti* Navone, Suriano & Pujol, 1990 were borrowed from the Harold W. Manter Laboratory of Parasitology (HWML, Lincoln, Nebraska); type specimens were deposited in the Colección Nacional de Helmintos (CNHE), of the Universidad Nacional Autónoma de México, México City. A single male collected from the examination of fluid preserved guts was deposited in the invertebrate collection of the AMNH.

**Table 1:** Information on gray mouse opossums and Mexican mouse opossums examined for pinworms. Specimens were collected in Oaxaca, Mexico and accessioned in the American Museum of Natural History, New York, NY.

Col. No.	Species name	Municipality	Locality name	Collector	Date	Sex	Pinworm infection
M-144638	*Tlacuatzin canescens*	Tehuantepec	Salazar	Thomas B. MacDougall	27-Feb-46	Male	Negative
M-165652	*Tlacuatzin canescens*	Tehuantepec	Las Tejas	Thomas B. MacDougall	20-Aug-50	Male	1 male
M-165653	*Tlacuatzin canescens*	Tehuantepec	Las Tejas	Thomas B. MacDougall	11-May-51	Male	Negative
M-189482	*Marmosa mexicana*	Pochutla	2 miles east of San Gabriel Mixtepec	A. L. Tuttle	25-Jul-62	Male	Negative
M-213651	*Marmosa mexicana*	Ixtlan	Santiago Comaltepec, Vista Hermosa	Thomas B. MacDougall	Not Available	Sex Unknown	Negative
M-232547	*Marmosa mexicana*	Ixtlan	Santiago Comaltepec, Vista Hermosa	Thomas B. MacDougall	27-Mar-69	Sex Unknown	Negative

**Table 2:** Measurements of type specimens used in the characterization and description of *Tlacuatzoxyuris simpsoni*, parasite of the gray opossum *Tlacuatzin canescens* (Allen, 1893) endemic to Mexico. Specimens were collected in the locality of Cruz Pintada, Morelos (18°27′58″N, 99°2′16″W, 1016 m), with the exception of AMNH IZC 331457, *Tlacuatzoxyuris* sp., from Las Tejas: Oaxaca (16°21′3.69″N, 95°20′3.86″W, 117 m).

Specimen	Allotype	Female 1	Female 3	Female 4	Female 5	Female 6	Female 7	Female 8	Female 9	Holotype	Male 2	Male 3	Male 4	Male 5	AMNH IZC 331457
Body length	2630	2427	2416	1715	1721	1452	1608	1619	1548	1477	1229		1216	1294	1023
Maximum width	163	170	201	123	138	131	138	111	194	155	107		113	145	130
Cephalic vesicle length	0	124	113	109	106	102	101		94	94	79	73	89	84	70
Cephalic vesicle width	68	69	61	59	66	59	58		60	65	50	55	54	63	49
Depth of stoma	5	8	5	9	6	8	7		6	–	9	7	7	7	3
Pharynx	–	–	–	–	–	–	4	8	10	–	11	13	8	11	9
Pharynx width	–	–	–	–	–	–	–	12	10	–	14	16	11	13	23
Esophagus length	256	250	251	220	225	216	231	237	220	–	186	196	175	189	179
Isthmus	13	10	10	13	13	16	16	20	11	–	13	9	11	11	9
			25	16	18	15	16	14	14	–	14	14	14	14	13
Corpus length	176	157	159	138	138	129	156	155	122	–	109	130	110	123	120
Corpus width	44	41	43	33	33	29	28	29	30	–	27	29	25	28	29
Bulb length	69	70	71	60	63	57	56	57	64	–	52	56	46	49	53
Bulb width	70	72	69	56	56	51	49	48	55	–	45	46	44	46	48
Distance anterior end to:															
Nerve ring	91	106	112	98	100	83	91	85	86	102	87	89	89	73	89
Excretory pore	504	452	480	351	337	322	341	469		418			324	342	286
Vulva	591	538	594	428	425	388	411	505	405						
Testis										570			472	474	280
Start area rugosa										718	710		629	569	537
Spicule length										90	88	84	81	88	65
Spicule width										5	6	5	6	5	
Gubernaculum										35	50	42	45	35	44
										5	4	4	5	4	5
Ovejector	87	86	–	–	–	–	–	–	–						
Post anal/cloacal terminus	166	348	415	363	372	245	271	347	413	52	46	45	42	45	31

## Results

### *Tlacuatzoxyuris* n. gen.

urn:lsid:zoobank.org:act:98673C5B-786D-4761-A6CE-87B1C2ECC83B

*Type species: Tlacuatzoxyuris simpsoni* n. sp.

*Etymology:* The genus name is a combination of the Nahuatl word *Tlacuatzin*, meaning opossum, and the composition *oxyuris* from the Greek words *oxys*, sharp and *oura*, tail.

*General:* Oxyuridae. Small worms, whitish when alive. Round cephalic plate, semicircular stoma, in terminal depression, surrounded by rim. Lateral alae emerge from cephalic vesicle and runs to tip of tail; alae show two crests.

*Male:* Prominent, round cephalic vesicle in males. Keel-like area rugosa present, with transverse striations. Four pairs of caudal papillae, two pairs adanal, one pair submedial, posterior to cloaca, one pair terminal. Rough cuticular plate immediately posterior to cloaca. Phasmids oriented dorsally. Spicule and gubernaculum present. Non-salient terminus.

*Female:* Cephalic cuticle excretory pore and vulva in anterior third of body; thick muscular vagina; didelphic.

### *Tlacuatzoxyuris simpsoni* n. sp.

urn:lsid:zoobank.org:act:2FA1FAC2-7A61-4E71-BBAC-B8072F07D2A0

(Figs. 1–12)

*Type host: Tlacuatzin canescens* (Allen, 1893).

*Symbiotype*: Universidad Autónoma de Morelos, Centro de Investigaciones Biológicas, Cuernavaca, Morelos (UAEM no number).

Type locality: Cruz Pintada: Morelos: Mexico (18°27′58″N, 99°2′16″W, 1016 m).

*Date of collection of type specimens:* 10 June 2010.

*Type-specimens deposited:* Holotype, male CNHE8347. Allotype, female CNHE8348; paratypes CNHE8349.

*Site of Infection:* Large intestine.

*Etymology:* The species is named after George Gaylord Simpson, American paleontologist who specialized in the study of mammalian evolution. His investigations on the Paleocene fossils from North America, as well as the Neogene fossils of Florida and Patagonia allowed him to formulate the mammalian transitions of the Great American Biotic Interchange.

**Figures 1, 2:**
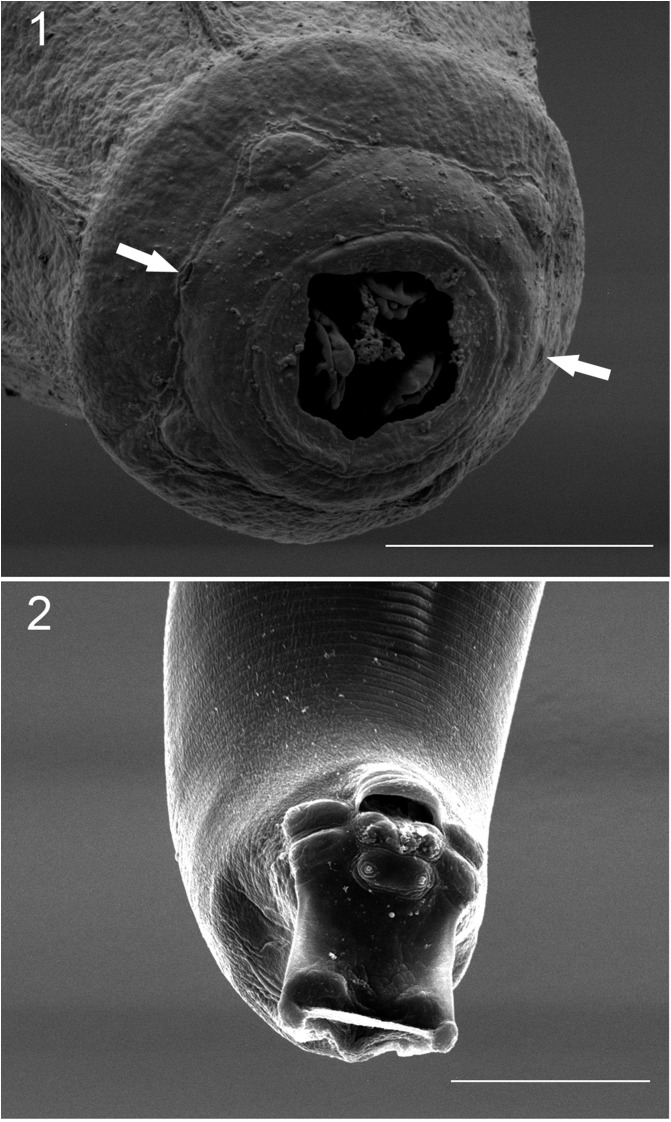
*Tlacuatzoxyuris simpsoni* n. gen. n. sp.*,* male. (1) cephalic plate showing relative placement of papillae, amphids (white arrows), rim around stoma and teeth; scale bar = 10 μm. (2) Ventral view of tail, showing paired papillae and postcloacal cuticular ornamentation; scale bar = 25 μm.

*General:* Small worms, whitish when alive; round cephalic vesicle, simple; body width gradually increasing posterior to cephalic vesicle, reaching maximum at midbody, and tapering towards posterior end. Round cephalic plate with four submedial papillae and lateral amphids (Fig. 1). Stoma semicircular, in terminal depression, surrounded by rim (Figs. 1 and 8). Esophagus endowed with three esophageal teeth (Figs. 1 and 8). Lateral alae emerge from cephalic vesicle and runs to tip of tail; alae taper at level of cloaca and anus in males and females, respectively. Alae show two crests (Fig. 4).

**Figures 3–6:**
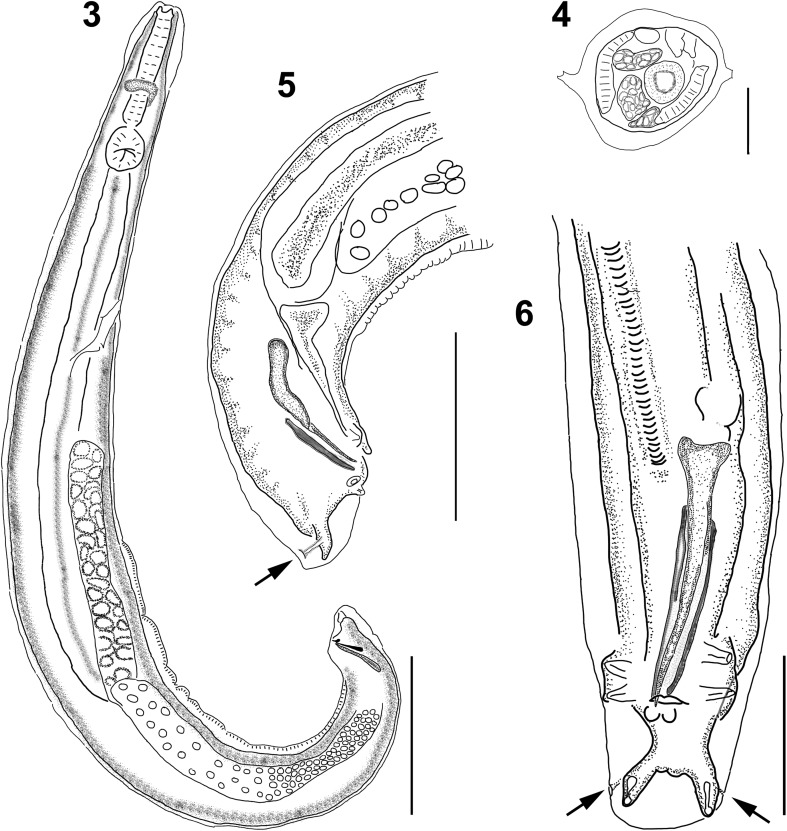
*Tlacuatzoxyuris simpsoni* n. gen. n. sp.*,* male. (3) lateral view of whole body; scale bar = 200 μm. (4) Cross section at midbody, showing double crested lateral alae; scale bar = 50 μm. (5) Lateral view of tail, arrow shows phasmid emerging from pedunculated papilla; scale bar = 100 μm. (6) Ventral view of tail, showing arrangement of papillae; scale bar = 50 μm.

*Males (based on five types, unless otherwise noted):* Small fusiform worms, length 1216–1477, 1304 (9%, *n* = 4) with both ends truncated (Fig. 3), maximum width at midbody 107–155, 130 (18%, *n* = 4). Cephalic vesicle 73–94, 84 (10%) long by 50–65, 57 (11%) wide. Well-developed lateral alae with two crests (Fig. 4), crests fuse into one at both ends; alae begin at level of nerve ring and ends anterior to cloaca. Esophagus length 175–196, 186 (5%, *n* = 4); corpus length 109–130, 118 (9%, *n* = 4), width 25–29, 27 (7%, *n* = 4); bulb length 46–56, 51 (8%, *n* = 4), width 44–46, 45 (2%, *n* = 4). Nerve ring, excretory pore and area rugosa located at 73–102, 88 (12%), 324–418, 361 (14%, *n* = 3), and 569–718, 656 (11%, *n* = 4) respectively from anterior end (Fig. 3). Continuous ventral area rugosa appears divided into 5–7 narrow mamelons (Fig. 3). Round postanal region 42–52, 46 (8%) long and 46–50, 48 (3%) wide (Figs. 2, 5, 6); distal end serves as basis for terminal papillae (Figs. 2, 5, 6). Non-salient terminus 28–38, 33 (13%) long (Figs. 5 and 6). Four pairs of genital papillae; two pairs sublateral, immediately anterior to cloaca; one pair subventral, postcloacal, and one pair subterminal, pedunculated (Figs. 2 and 6). Postcloacal ornamentation in narrow area between cloaca and subventral papillae (Fig. 2). Phasmids emerge from the peduncle of terminal papillae towards dorsal side (Figs. 5 and 6). Monorchid. Spicule with moderate inflection, length 81–90, 86 (4%), width at calamus 5–6, 5 (11%). Gubernaculum 35–50, 41 (16%) long, 4–5, 4 (8%) wide (Figs. 5 and 6).

**Figures 7–12:**
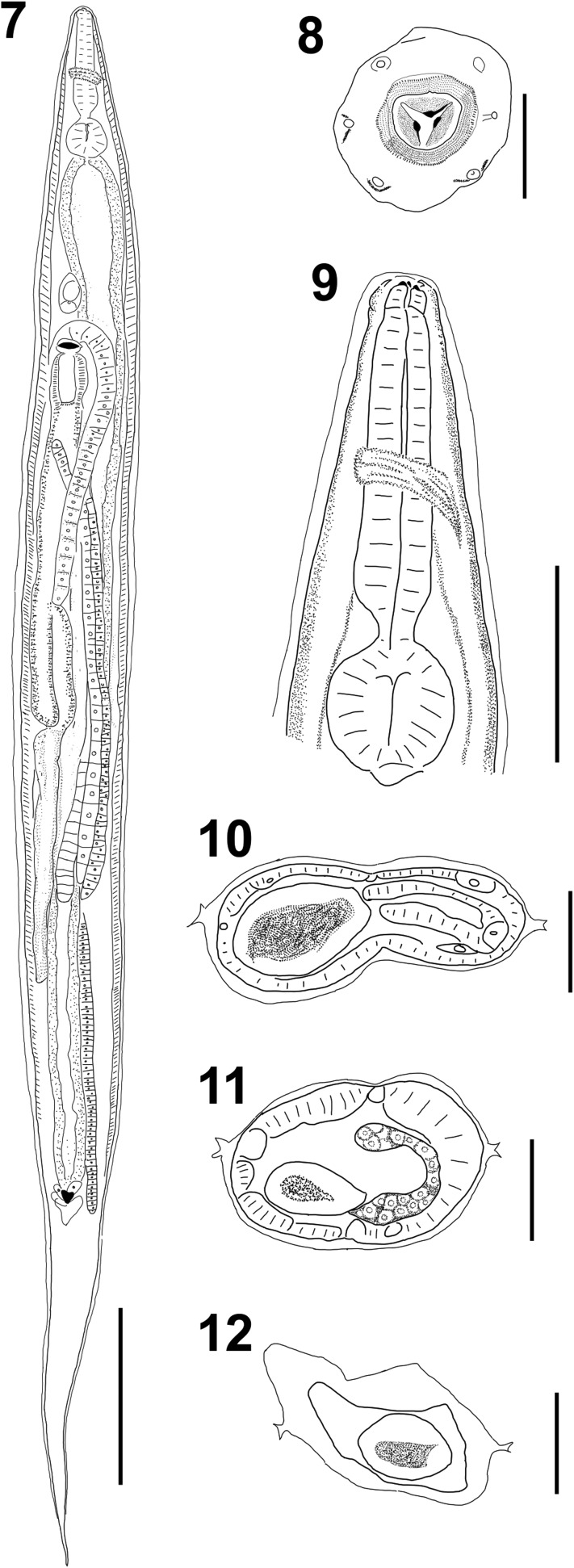
*Tlacuatzoxyuris simpsoni* n. gen. n. sp.*,* female. (7) Ventral view of whole body; scale bar = 300 μm. (8) Face view, showing teeth and continuous cuticle surrounding stoma; scale bar = 20 μm. (9) Esophagus, showing relative position of nerve ring, scale bar = 100 μm. Cross sections at level of (10) vulva, (11) midbody, and (12) slightly anterior to anus; scale bar = 50 μm.

*Females* (*based on nine types, unless otherwise noted*)*:* Fusiform worms, length 1452–2630, 1904 (24%), maximum width at midbody 111–201, 152 (21%) (Fig. 7). Cephalic vesicle 94–124, 107 (9%, *n* = 4) long by 56–69, 62 (7%, *n* = 4) wide. Lateral alae emerge from cephalic vesicle, runs continuously to tail end; two crests at level of vulva, midbody and rectum (Figs. 10–12). Esophagus length 216–256, 234 (7%); corpus length 122–176, 148 (12%), width 28–44, 35 (19%); bulb length 56–71, 64 (9%), width 48–72, 58 (16%) (Fig. 9). Nerve ring, excretory pore and vulva located 83–112, 95 (11%); 322–504, 407 (19%) and 388–594, 476 (17%) respectively from anterior end (Fig. 7). Vulva connected to muscular ovejector, ovejector to short vestibule and this to two uterine branches (prodelphic). Muscular ovejector 74–87, 82 (9%) long (Fig. 7). Tail tapers to a point, 165–415, 326 (25%) long (Fig. 7). Eggs not observed.

### *Tlacuatzoxyuris* sp.

*Host: Tlacuatzin canescens* (Allen, 1893).

*Host deposition:* AMNH165652, Mammalogy.

*Locality:* Las Tejas: Oaxaca: Mexico (16°21′3.69″N, 95°20′3.86″W, 117 m), collected August 20, 1950.

*Specimens deposited:* 1 male AMNH IZC 331457.

*Site of Infection:* Large intestine.

*Male:* Small worm, round cephalic vesicle, simple; body width gradually increasing posterior to contracted cephalic vesicle, reaching maximum at midbody, and tapering towards posterior end. Round cephalic plate. Lateral alae run to tip of tail; alae taper at level of cloaca. Length 1023 with both ends truncated, maximum width at midbody 130. Cephalic vesicle 70 long by 48 wide. Alae begin at level of nerve ring and end at level of most posterior mamelon. Esophagus length 179; corpus length 120, width 29; bulb 53 by 48. Nerve ring, excretory pore and area rugosa located at 89, 286 and 228 respectively from anterior end. Continuous ventral area rugosa appears divided into six narrow mamelons. Round postanal region 31 long and 50 wide. Non-salient terminus nine long. Four pairs of genital papillae with pedunculated subterminal pair. Monorchid. Spicule with moderate inflection, length 64. Gubernaculum 44 long.

## Discussion

The genus *Tlacuatzoxyuris* can be discriminated from other genera present in the northern Neotropics by the absence of an external terminus in the tail of males and a prominent caudal ala. It can be discriminated from *Helminthoxys* Freitas, Lent and Almeida, 1937, because *Tlacuatzoxyuris* feature a cephalic vesicle with no prominent cervical alae; from *Heteromoxyuris* Quentin, 1973 because males feature 5–7 mamelons as ventral ornamentation, and from *Passalurus* Dujardin, 1845 in that males are endowed with a spicule. Further, the new genus can be discriminated from members of *Trypanoxyuris* Vevers, 1923 because species in the latter feature complex labial and buccal structures and no ventral mamelons. Finally, the new genus can be discriminated from the five genera of oxyurids present in Australian marsupials (*Austroxyuris* Johnston & Mawson, 1938; *Adelonema* Mawson, 1978; *Paraustroxyuris* Mawson, 1964; *Macropoxyuris* Mawson, 1964 and *Potoroxyuris* Mawson, 1964) in that males included in those taxa have relatively more complex and deep buccal capsules, prominent caudal termini, a caudal ala, and a cuticle with very few mamelons or no ventral ornamentation in males.

*Tlacuatzoxyuris simpsoni* can be discriminated from all other four species of pinworms infecting mouse and fat-tailed opossums based on the configuration of the stoma and the relatively short spicule and gubernaculum. The stoma of *T. simpsoni* is separated from the cephalic plate by a terminal rim, it is in a shallow terminal depression and it has the appearance of a very irregular semicircular structure (Figs. 1 and 8). The postcloacal cuticle of males shows ornamentation immediately posterior to the cloaca, just anterior to the pair of submedial papillae (Fig. 2). Finally, both spicule and gubernaculum are relatively short. The eggs of *Tlacuatzoxyuris* remain unknown.

**Figure 13:**
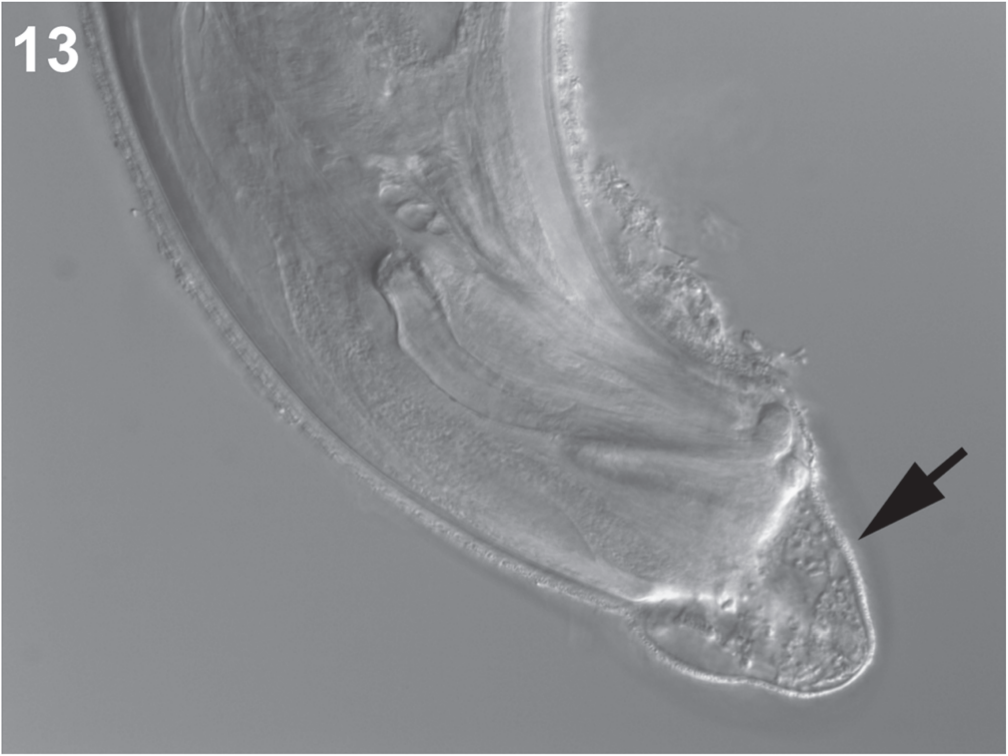
Male, *Didelphoxyuris thylamisis*, showing extension of postcloacal ornamentation.

*Tlacuatzoxyuris simpsoni* can be differentiated from *D*. *thylamisis* based on four main characteristics. First, the relative and absolute size of the spicules and gubernaculum are larger in *D*. *thylamisis* than in the homologous structures in *T*. *simpsoni*. Second, the cuticle immediately posterior to the cloaca of *T. simpsoni* has ornamentation limited to a small patch between the cloaca and the submedial papillae (Fig. 2), whereas tubercle-like bumps are present on the surface of the cuticle posterior to the submedial papillae of *D*. *thylamisis* (Fig. 13)*.* Third, the cephalic vesicle of females of *T. simpsoni* is not very well developed and the cuticle is simple, lacking the characteristic complex reticulated network of vesicles seen in the type specimens of *D. thylamisis*. Finally, the stoma of *T. simpsoni* is subterminal and surrounded by an irregular cuticle (Figs. 1 and 8), whereas the homologous structure appears to be depressed, covered by cuticular flaps and endowed with esophageal teeth in *D. thylamisis* (see Figs. 1B, C, and 4A, C in Gardner and Hugot [12].

*Tlacuatzoxyuris simpsoni* can be readily distinguished from *M. dollmeiri* in that the latter bears a prominent caudal terminus that emerges from the cuticle, and because the cuticle surrounding the stoma features three lips. The terminus in *T. simpsoni* is non-salient, a trait shared with the other three monotypic genera. The species described herein features three esophageal teeth, an arrangement that contrasts with the four teeth described in *N. venusti*. In addition, males of the latter species feature up to six pairs of small precloacal papillae. Finally, *T. simpsoni* can be discriminated from *G. agilisis* in that the stoma of the latter presents three or more divisions described as lips, as well as a weakly developed cephalic vesicle. The divisions around the stoma appear to be cuticular flaps that cover the stoma. These are divided in some of the specimens observed with the aid of SEM [8, 24].

For didelphimorph-dwelling pinworms, the ornamentation of the eggs has been used as an important characteristic to justify the placement of species in monotypic genera. In particular, eggs in *Didelphoxyuris* are described as non-operculated, whereas the eggs of *Gracilioxyuris* show three longitudinal ridges with an anterior operculum. This character is of paramount importance in the definition of *Gracilioxyuris* [8, 24], since both *Gracilioxyuris* and *Didelphoxyuris* share similarities on the structure of the cephalic and caudal arrangement of papillae.

No gravid female was collected as a result of our efforts, which yielded a single male from the three screened gray opossums from Tehuantepec and only mature females from the type locality. We predict that the eggs for this species will be operculated as is the case in eggs of *M. dollmeiri*, *N. venusti* and *G. agilisis*. A taxonomic revision based on a reconstruction of the relationships among the opossum-dwelling pinworms is necessary to clarify homologous characters defining the monotypic genera.

The single specimen collected from Oaxaca likely belongs to a different species, based on the characteristics of the spicules, distance of the area rugosa to anterior end and the shape of the cephalic vesicle. However, the characterization of the species requires a description of character variability of the cephalic plate and vesicle, the arrangement of the caudal papillae and configuration of the structures present in females. Thus, we are unable to present a description at this time. Interestingly, the presence of this second pinworm species mirrors the splitting of the marsupial host lineage [3]. The pinworms herein documented infected individuals collected from two different clades that represent the geographic clusters of the Balsas basin and the Tehuantepec Isthmus.

Because there is a bias towards the study of common species, we urge colleagues to document the diversity of metazoan parasites associated with all didelphimorph marsupials. The greatest species diversity of didelphimorph marsupials includes small sized opossums, most of which are arboreal or are restricted to small areas in the tropical forests [4, 6]. We urge the examination of preserved mammalian guts available in scientific collections to continue reconstructing the biological inventory of their symbionts, which includes parasites. In addition, we recommend that scientists working in the field should preserve the parasites or the tissues of freshly caught mammals in the field and link both parasite and host through a relational database [10]. The completion of this parasite inventory will give us an opportunity to better understand the expansion of the marsupials and their pathogens as they were both involved in the Great American Biotic Interchange and may be involved in subsequent events of dispersion or translocation.
